# Understanding Patient Registries for Diabetes: A Scoping Review of Published Literature

**DOI:** 10.1177/23743735251314620

**Published:** 2025-01-21

**Authors:** Lana Moayad, Paige Alliston, Saira Khalid, Donna Fitzpatrick-Lewis, Hertzel C. Gerstein, Diana Sherifali

**Affiliations:** 1 Faculty of Health Sciences, Department of Medicine, McMaster University, Hamilton, Canada; 2 Faculty of Health Sciences, School of Nursing, 3710 McMaster University, Hamilton, Canada; 3 Population Health Research Institute, Hamilton Health Sciences, 3710 McMaster University, Hamilton, Canada; 4 Diabetes Care and Research Program, The Boris Clinic, 3710 McMaster University Medical Centre, Hamilton Health Sciences, Hamilton, Canada

**Keywords:** diabetes, patient engagement, communication, community engagement

## Abstract

**Background:** Diabetes registries have grown in prevalence and incorporated patient engagement opportunities to support diabetes management. We aimed to understand the goals, purpose, and context for diabetes registries defined as patient-focused and how people with diabetes are engaging with these registries. **Methods:** We searched Pubmed, MEDLINE, Embase, and Emcare using the following criteria: (1) the population is people with diabetes mellitus, including type 1, type 2, and/or gestational diabetes; and (2) the study describes a patient focused registry. **Results:** The search identified 346 citations, 9 of which were included. The goals of the registries included: developing referral systems, evaluating community-based interventions, collecting self-reported data, improving access to care, and fostering diabetes communities. The delivery settings were community-based, outpatient, or primary care. The methods of delivery and level of patient engagement varied between registries. **Conclusions:** This scoping review identified 9 diabetes registries, with varying goals, purposes and levels of patient engagement. It highlights a need for registries centered on people with diabetes to promote engagement and facilitate long-term diabetes self-management.

## Key Messages

1. Our review identified 9 diabetes registries.
2. Although the 9 registries involved some degree of patient engagement, there were varying levels of engagement throughout.
3. The purpose of patient registries aligned more with population health surveillance and clinical care, rather than building a diabetes community.
4. Peer-reviewed literature to date highlights a paucity of patient engagement in patient registries for diabetes.

## Introduction

Over 10% of Canadians are currently diagnosed with diabetes, including type 1, type 2, and gestational diabetes.^1^ This number is projected to rise worldwide, reaching over 578 million by 2030.^2^ As a tool for monitoring and surveillance, patient registries can be used to collect and store data relevant to patient outcomes.^3^ Although there is no consistent definition of a patient registry,^4^ a patient registry involves using an organized system to facilitate the collection of data on a specific population of individuals that can be used to evaluate certain outcomes to support a disclosed purpose (ie, research, policy, clinical, or other).^5,6^

There are currently hundreds of patient registries globally that contribute to and advance existing health systems.^7^ In the diabetes community, patient registries have become more prevalent in recent decades.^8^ Numerous countries have employed diabetes registries to survey the prevalence of diabetes and monitor access and quality of diabetes services.^3^ In most cases, national diabetes registries recruit individuals directly through administrative health databases and/or electronic medical records (EMRs) via clinical healthcare settings, with some registries seeking individuals through physician referrals.^9^ Similar to the limitations of patient registries globally,^10^ most diabetes registries do not allow individual patients to access their information or inform their care.^9^ Examples of such large diabetes registries include the Diabetes Collaborative Registry in the United States, the National Diabetes Register (NDR) in Sweden, the Diabetes-Patienten-Verlaufsdokumentation (DPV) in Germany, and the National Diabetes Services Scheme (NDSS) in Australia.

Of the 12 largest national diabetes registries identified in a recent systematic review, only the NDSS provides diabetes self-management support resources to registry participants.^9,11^ These support resources include practical help and guidance, diabetes health information, and subsidized diabetes products.^11^ Providing self-management education and support for people living with diabetes is recommended,^12,13^ as self-management supports the optimization of diabetes control and the prevention of complications.^14^ Additionally, only 3 registries provide an avenue to collect self-reported outcome measures (the Norweigan Diabetes Register for Adults [NDR-A], Swedish NDR, and German DPV), with the NDR-A purposefully recruiting participants to report on topical and timely concerns such as COVID and diabetes distress.^9,15,16^ The Swedish NDR, particularly, supports individuals to actively monitor their data, interact with healthcare providers, and participate in shared decision-making.^17^ A similar trend is seen in pediatric diabetes registries, with the Swedish Childhood Diabetes Registry being one of the only registries to provide participants or their families access to their information.^17^

Increased patient engagement within patient registries indicates the beginning of a shift to understand better and meet the needs of individuals with diabetes rather than monitoring clinical outcomes alone.^7^ As described by Abelson et al,^18^ patient engagement can be defined as a “meaningful and active collaboration in governance, priority setting, conducting research and knowledge translation” (p. 5). In alignment with goal-oriented care, patient engagement allows for data collection and monitoring within the registry to reflect what is most meaningful to patients.^10^ Various frameworks have been developed to communicate levels of patient engagement, with one combined framework describing 3 levels of shared leadership between researchers and their patients. This engagement typology emphasizes a partnership between researchers and their patients in the exchange of information, consultation, or participation.^18–20^ Patient engagement continues to evolve^21^ to provide individuals with diabetes with the ability to connect with peers, practice advocacy and self-expression, and share information.^22^ Patient registries may offer an opportunity to merge the collection of clinical and self-reported data with patient engagement to share evidence-based information on diabetes management and facilitate positive connections with peers and the registry curators.

Considering the known utility and future opportunities of patient registries, we seek to understand what patient registries currently exist that emphasize patient engagement and are led or operated with the support of patient communities, rather than a database of patient-derived data only. We conducted a scoping review of peer-reviewed published literature to identify patient-focused (eg, patient-led, patient-operated, or patient-accessed) registries globally. We aimed to understand the goals, purpose, and context for diabetes registries defined as patient-focused and how patients are engaged with these registries.

## Method

To begin to understand the ambiguity of the literature and the lack of definition of the term “registries,” we used a scoping review approach to understand the breadth of the literature on this topic.^23^ A scoping review allowed us to conduct a broad literature view, include a variety of study designs, and explore the use of patient registries in multiple diabetes-related settings. We followed the PRISMA checklist for scoping reviews throughout our review.^24^

### Search Strategy

Broadsearch terms, databases, and strategies were developed in consultation with a research librarian (Appendix 1). We searched Pubmed, MEDLINE, Embase, and Emcare from inception to April 16, 2023. Results from the search were deduplicated, and citations were uploaded to a secure internet-based platform for screening (DistillerSR, Evidence Partners Inc., Ottawa, Canada). Eligible studies of any design/type had to be published in English in a peer-reviewed journal and meet the following criteria: (1) the *population* of interest is people with diabetes mellitus, including type 1 diabetes, type 2 diabetes, and/or gestational diabetes; and (2) the study describes a registry that is patient-focused (eg, patient-led, patient-operated, or patient-accessed). Studies where the registry was a main component of a trial (ie, used for recruitment or to support intervention delivery) or a secondary analysis were also included. Outcomes were not used to include or exclude studies. Studies were excluded if they described a physician-driven exclusively, and/or genetic database registry.

### Literature Selection

A team of researchers conducted the screening and data extraction. A minimum of 2 reviewers were required to independently and in duplicate screen titles and abstracts of all potentially eligible studies. Articles marked for inclusion by either team member went on to full-text screening which was completed independently and in duplicate by 2 team members and required consensus for inclusion or exclusion. Conflicts were resolved through discussion with the lead researchers of this review. After confirmation of the included studies, we looked for related publications that met our search dates and inclusion criteria and grouped multiple publications that were based on the same study/intervention.

### Data Extraction

**Figure 1.**
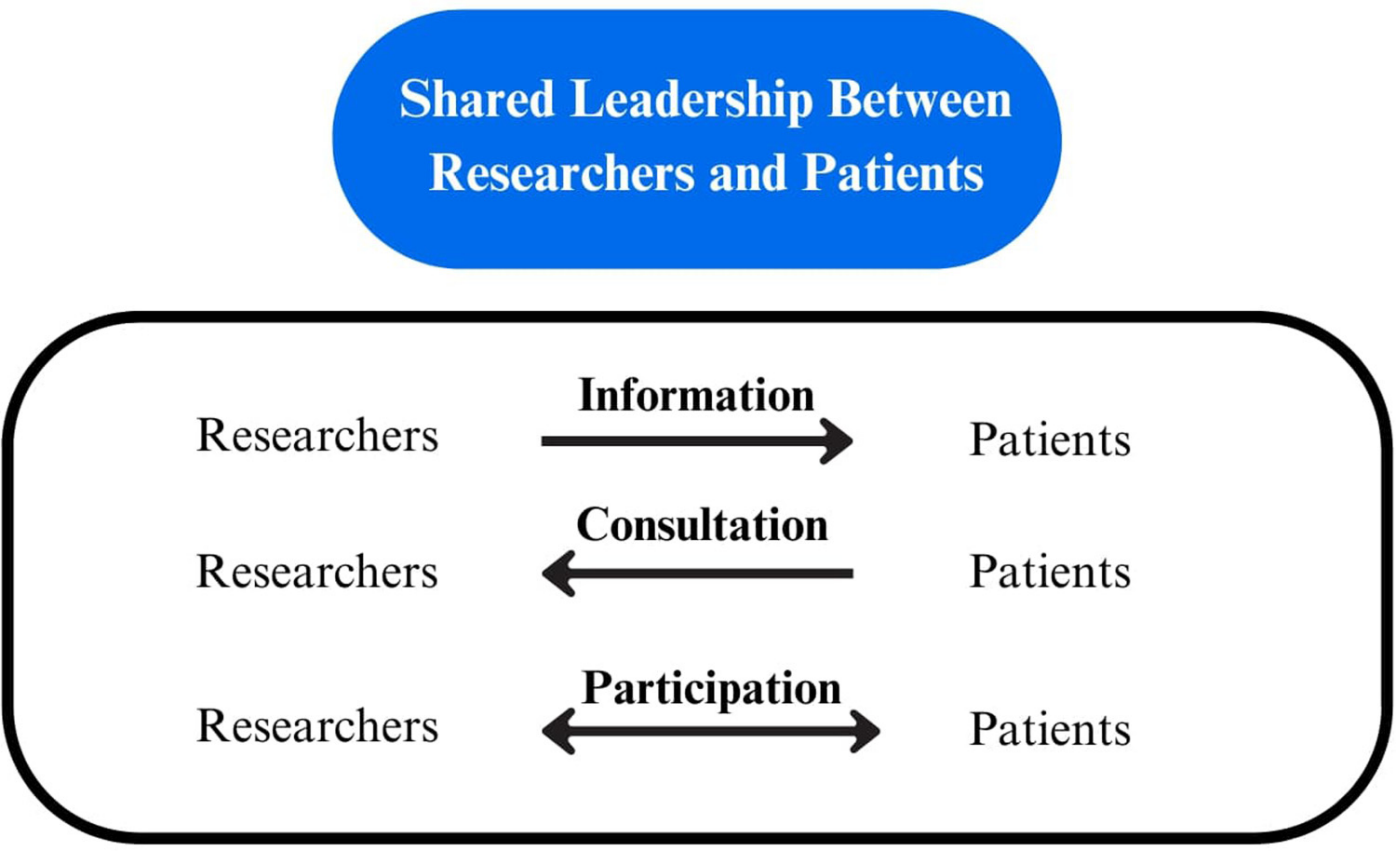
Patient engagement framework adapted from Abelson.^18^

We developed, piloted, and deployed standardized forms for data extraction which were housed in a web-based systematic review software program (DistillerSR). All authors provided feedback and approved the components of these forms. For each primary study, 1 team member extracted study characteristics (including the country of origin, healthcare setting, aim of study, aim of registry, sample size, study design, and registry methodology) and the patient, disease, and provider characteristics included in the studies. We applied the engagement framework by Abelson to understand the level of patient engagement occurring within each registry.^18^ This typology describes an exchange of information, consultation, or participation between researchers and patients, with shared leadership occurring at each level of engagement. This framework is highlighted in Figure 1 and applied to each of the included studies. Two team members independently verified all extracted data and disagreements were resolved through discussion and/or third-party consultation.

## Results

**Figure 2.**
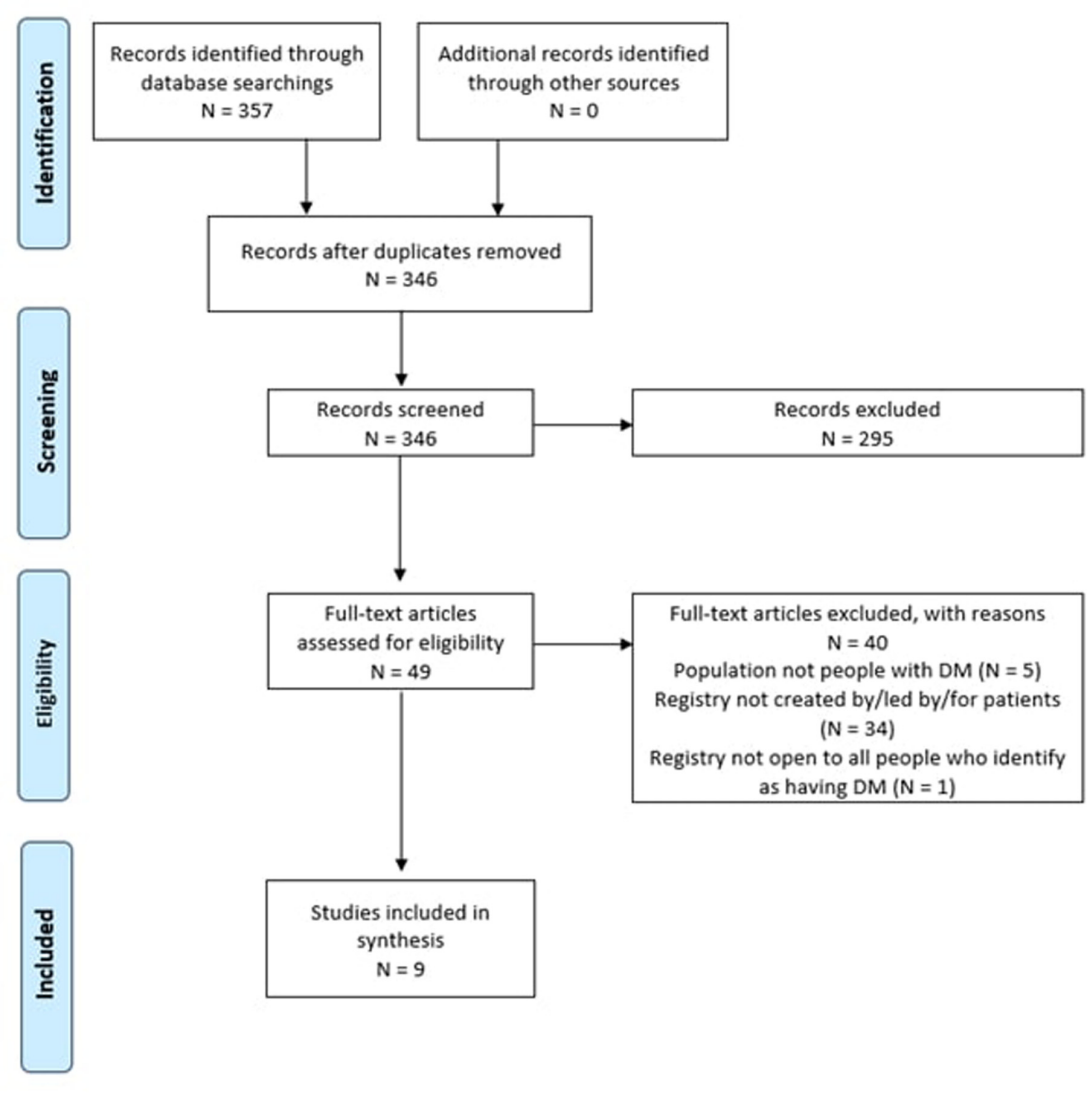
PRISMA flowchart of included studies.

The search identified 346 citations to be assessed after the removal of duplicates. A total of 49 full-text articles were assessed for eligibility and 40 were excluded due to: (a) not being created or facilitated by or for patients with diabetes; or (b) not including patients with diabetes. The final group of studies (*n* = 9) was included in our scoping review.^25–33^ Figure 2 summarizes the study selection process for this review.

### Characteristics of Included Studies

The group of included papers was mostly retrospective cross-sectional studies,^25–27,30,32,33^ with some being randomized controlled trials,^28,29^ and 1 study testing the piloting phase of their registry.^31^ There were 4 registries based out of the United States,^27,29,31,32^ 2 registries from Canada,^30,33^ and 1 registry from Australia,^28^ Israel,^26^ and Germany,^25^ respectively. The study from Germany included a registry that was a subsample of a larger network of registries across Germany, Austria, Switzerland, and Luxembourg.^25^ The registries were developed and utilized by researchers in multiple settings, including community-based,^31–33^ outpatient,^25,27^ and primary care settings.^26,29^ The number of participants within the registries varied from 19 patients for a study pilot testing a registry^31^ to 3,936,800 patients for a government registry actively running for 12 years.^26^ The goals of the registries were diverse and included: evaluating patient characteristics (blood pressure, lipids, physical activity),^26,31,34^ monitoring diabetes populations using EMR data^25^ developing a standardized referral and follow-up system for primary care physicians using EMR data,^26^ evaluating community-based^28^ and clinical interventions,^29^ improving access to care and patient empowerment,^32^ and fostering diabetes friendly communities.^33^ Some of the registries were described as web-based^30,32,33^ with data being submitted and stored electronically, and 1 registry offered opportunities to attend in-person educational events as a member of the online patient registry.^33^ Patients were recruited through flyers, letters, emails, events, multimedia, social media, or in-person after a follow-up appointment.^30,33^ Healthcare providers were also invited to join certain registries to encourage their patients to practice self-management behaviors.^33^ A summary of characteristics of the included studies can be found in Table 1, with detailed characteristics found in Supplemental Table S1.

**Table 1.** Characteristics of Included Studies.

Author(s), Year	Country of origin/healthcare setting (context)	Goal/purpose of registry	Patient engagement	Sample size	Inclusion/exclusion	Registry methodology
^25^	Germany/300 diabetes centers	NR	NR	Registry from 300 German centers: 32,785 total sample in diabetes centers from Germany, Austria, Switzerland, and Luxembourg: 644,720	Inclusion: electronic health record developed at the Institute of Epidemiology and Medical Biometry, Ulm University, Germany	NR
^26^	Israel/Primary care clinics and hospitals (Clalit)	The main infrastructure changes were allowing primary care physicians to refer patients for hemoglobin A1c (HbA1c) and low-density lipoprotein, inserting standardized follow-up forms for diabetes patients, establishing a computerized diabetes registry (first at the clinic level and later at the national level), and developing standardized reports regarding the quality of diabetes follow-up and control within the electronic medical record. To compare process and outcome measures among Clalit's members with diabetes, to diabetes patients enrolled in a different health maintenance organization (LHS). A similar registry of diabetes patients was also developed in LHS in order to facilitate reporting to the National Program for Quality Indicators of Health in Israel.	Level of engagement: information access to educational material (self-care instruction kit, video, website cookbooks), lifestyle improving workshops	Clalit Health Services: 3 936 800 LHS: 692 500	Inclusion: diabetes patients in Clalit	NR
^27^	USA/urban outpatient diabetes clinic	NR	NR	345	Inclusion: included in a computerized registry; had a 1-year (52 + 10 weeks) follow-up visit; if serum total cholesterol, HDL cholesterol, LDL cholesterol, and triglyceride levels were measured at both the initial (baseline) and 1-year visits.	NR
^28^	Australia/community-based	Administered by Diabetes Australia, NDSS is an initiative of the Australian Government to provide diabetes-related information and support services to people living with diabetes	Level of engagement: information access to diabetes health information, programs, and subsidized diabetes products	273	NR	NR
^29^	USA/primary care	NR	NR	87	NR—the registry was used as a recruitment method for the RCT	NR
^30^	Canada/community-based (Quebec)	The BETTER (BEhaviors, Therapies, TEchnologies and hypoglycemic Risk in type 1 diabetes) registry is a type 1 diabetes population surveillance system to address the burden of hypoglycemia and assess the impact of new therapies and technologies.	Level of engagement: participation codevelopment of the registry (patient partners), access to registry materials (website)	1430	Inclusion: individuals must self-report a clinical diagnosis of T1D or latent-autoimmune diabetes in adults (LADA), provide a valid address in the province of Québec (Canada) and be able to read French or English	Recruitment methods: (1) research personnel, (2) poster/flyer, (3) letter/email, (4) social media, (5) diabetes organizations, (6) healthcare professionals, (7) patient conferences, (8) diabetes events, (9) study website, (10) Connect1D platform, (11) patient partners; delivery methods: online questionnaire; main outcomes of the registry; clinical versus lifestyle data; patient-reported data
^31^	USA/NR	NR	NR	19	NR—the registry was a recruitment method for the pilot intervention study	NR
^32^	USA/community-based (Chicago)	The web-based patient registry, the BridgingCare Planner, is used to generate a list of patients with diabetes, record patient attendance to DRIVE day, and track clinical data to support the Austin Health Center in monitoring key diabetes indicators.	NR	294	NR	Delivery methods: web-based
^33^	Canada, community-based (Hamilton)	DH's goals have been to: (a) supplement the limited diabetes specialty services and tools by identifying, developing and providing community-based resources; (b) promote and facilitate diabetes self-management by a broad range of health care providers; (c) create a “diabetes friendly” community; and (d) sensitize the city in general to the growing diabetes epidemic	Level of engagement: participation Receiving community-based resources (newsletter, resource inventory, website, interacting with diabetes community (website, educational events)	Individuals: 3161; family physicians: 550	Inclusion: people with diabetes and healthcare providers in the Hamilton area	Recruitment methods: personal invitations, multimedia promotion (TV), radio, and newspaper, public education events Delivery methods: in-person, web-based Main outcomes of the registry: clinical versus lifestyle data; patient-reported data; frequency of data collection and reporting: baseline, annually

NR, not reported.

*Further details available in Supplemental Table.

### Characteristics of Included Patients and Participants

Data on the characteristics of patients was collected within the registries for 7 of the 9 included studies.^25–27,29,30,32,33^ Specifically, the registries of these studies collected information on gender,^25–27,30,32,33^ age,^25–27,29,30,32,33^ race/ethnicity,^25,27,29,30,32,33^ location,^25,26,30,33^ and social determinants of health^26,30,32,33^ such as insurance type, education, and location. None of the included studies collected data on the characteristics of healthcare providers. Seven of the studies incorporated diabetes-related patient information within the registry.^25–27,29,30,32,33^ This included years of diagnosis and type of diabetes. Six studies report capturing HbA1c.^25–27,29,30,32^ Three studies also collected information on comorbidities^28,33^ and 5 reported self-management behaviors within the registry.^25,27,30,32,33^ A detailed outline of patient, provider, and disease characteristics within the registries can be found in Table 2.

**Table 2.** Characteristics Within the Registry.

Author, year	Patient characteristics collected in registry	Disease characteristics collected in registry	Provider characteristics collected in registry
^25^	Race/ethnicity; gender; age; location	Diagnosis/diabetes duration; type of diabetes; lab values: BG, A1C; Management: pills, injectables, insulin, procedures, lifestyle modification	NR
^26^	Gender; age; location (% % rural vs Arab settlements); SDH (% receiving welfare, handicapped, hired workers; wages (% minimum or above average))	Diagnosis; comorbidities; lab values: HbA1c, LDL, microalbumin	NR
^27^	Race/ethnicity; gender, age	Diagnosis/diabetes duration; type of diabetes; lab values: HbA1c, total cholesterol, LDL, TG, HDL; Management: mode of treatment for hyperglycemia	NR
^28^	NR	NR	NR
^29^	Race/ethnicity; age	Diagnosis (years since); comorbidities; lab values: HbA1c	NR
^31^	NR	NR	NR
^30^	Contact information (email); name; date of birth; race/ethnicity; gender; age; language (English, French); location: SDH: household income, education, employment status, insurance status	Diabetes duration (years); type of diabetes (T1D, LADA); diabetes-related complications (cardiovascular disease, nephropathy, neuropathy, retinopathy, gastroparesis); lab values: A1C; management: current treatment modalities, commonly used medications, management and consequences of hypoglycemia, diabetes treatment, fear of hypoglycemia, diabetes distress, IAH (Clarke score) and some lifestyle habits (smoking status and use of alcohol/drugs), hypoglycemia treatment, management of hyperglycemia, sleep habits, stigmatization, barriers to physical activity and depression	NR
^32^	Contact information (phone number); name (patient identification for attendance at DRIVE day); race/ethnicity; gender; age; SDH (insurance type)	Comorbidities; foot and eye examination; lab values: A1C, LDL, SBP, DBP; management: medication; education on diabetes, nutrition, exercise	NR
^33^	date of birth; race/ethnicity; gender; age; location (postal code); SDH: education	Diagnosis/diabetes duration; comorbidities; management: self-care behaviors, risk factor screening, medication use, lifestyle behaviors	NR

NR, not reported.

### Degree of Patient Engagement

Patient engagement in the included registries was described in 4 studies.^26,28,30,33^ The degree of patient engagement varied, with 2 registries classified under the “participation” level of the patient engagement framework.^18,30,33^ One of these registries was codeveloped with patient partners^30^ and another conducted a needs assessment with relevant stakeholders, including patients ahead of development.^33^ The 2 registries classified under the “information” level of patient engagement^18^ utilized the registries in different ways, including accessing subsidized products (eg, coupons for devices)^28^ and attending events and workshops offered through the registry.^26^ Resources available for patients within these registries include self-care kits, videos, and cookbooks,^26^ educational websites,^26,30,33^ links to self-management services,^28^ and newsletters.^33^ An overview of patient engagement is summarized in Table 1.

## Discussion

This scoping review aimed to assess the current state of patient diabetes registries. Our findings demonstrate the diversity of 9 diabetes patient registries of varying goals and purposes, that claim to be “patient-focused” in development and implementation. For multiple studies, the purpose of the registry was not disclosed.^25,27,29,31^ Through our scoping review approach, we included studies where the diabetes registry was a main component of a trial or a secondary analysis to highlight the different uses of patient registries across the diabetes landscape. Communicating the purpose of a registry is an important component of registry development, as it allows the data collection to be purpose-driven, identifies important stakeholders,^4^ and supports the ability to obtain funding.^35^ For the included papers that did describe the purpose of their registries^26,28, 30^^,^^32^^,^^33^ the variety of rationales behind creating a registry (ie, surveillance, clinical care, program evaluation, and community development) attests to how different stakeholders, including individuals with diabetes, utilize patient registries and maximize their value.^4^

We also included registries where patient engagement was mentioned but was not the main focus of the registry. This emphasizes the discrepancies that currently exist in diabetes patient registries that report on patient engagement. Two of the included studies collected information from electronic health records (EMRs)^25,26^ rather than including an element of active data collection. Linking primary and secondary sources of data can help to enhance a registry and open up opportunities to explore primary and secondary objectives.^4^ However, depending on the purpose of the registry and available funding, the information sources utilized in a registry can be customized to address specific research questions.^5^

The degree to which patients were actively participating in engagement with researchers was reported in 2 of the included studies, with 1 study conducting a needs assessment with diabetes stakeholders (including those living with diabetes) ahead of inception,^33^ and another study codesigning the registry with patient partners.^30^ Involving participants in active participation at enrollment increases the likelihood of ongoing participation for the life of the registry.^4,5^ For registries including clinical data for individuals accessing care from multiple sources, or patient-reported outcome data, incorporating a plan that follows participants across their circle of care is recommended.^5^ These 2 studies also relied on self-reported data to gather information from participants,^30,33^ emphasizing the knowledge transfer that was actively occurring between researchers and patients.^18^

## Limitations

Strengths of our review include our rigorous search strategy, strict inclusion criteria, and strong methodological approach to comprehensively assess the current literature on diabetes registries. A limitation of our review is that we only searched published literature. Although unlikely, we recognize that the existence of patient registries that enhance patient engagement may be available and could be assessed through internet searches. Additionally, we conducted a scoping review to explore this topic with an understanding that the term “registry” is not clearly defined. Our search strategy was broad and our inclusion criteria allowed us to capture the varying definitions and uses of registries in the diabetes context, which led to the inclusion of studies that did not describe the collection of data or omitted information that would help us better understand the level of patient engagement within the registry.

Our group aims to build on the findings of this review by developing a patient-led and reported diabetes registry with a focus on patient engagement, specifically for information and participation (eg, knowledge transfer).^18^ The registry will provide an avenue for participants to engage with researchers, and access evidence-based information and resources on diabetes management. In alignment with the patient engagement framework,^18^ registry participants will be actively involved in shared leadership within the registry and will contribute to an ongoing cycle of knowledge transfer. This will occur through self-reported data, feedback, and input in what information is offered to patients within the registry.

## Conclusion

The results of our scoping review identified heterogeneity in the definition of patient-focused registries, goals, and purposes, with some registries providing limited patient engagement opportunities. As patient registries continue to evolve globally to integrate active participation,^7^ our findings support the need for future patient registries to be developed that incorporate patient engagement from registry development to the ongoing management of the registry. Increased engagement will support individuals living with diabetes in monitoring information that is most relevant to them and adhering to long-term self-management.

## Supplemental Material

sj-docx-1-jpx-10.1177_23743735251314620 - Supplemental material for Understanding Patient Registries for Diabetes: A Scoping Review of Published Literature Supplemental material, sj-docx-1-jpx-10.1177_23743735251314620 for Understanding Patient Registries for Diabetes: A Scoping Review of Published Literature by Lana Moayad, Paige Alliston, Saira Khalid, Donna Fitzpatrick-Lewis, Hertzel C. Gerstein and Diana Sherifali in Journal of Patient Experience

sj-docx-2-jpx-10.1177_23743735251314620 - Supplemental material for Understanding Patient Registries for Diabetes: A Scoping Review of Published Literature Supplemental material, sj-docx-2-jpx-10.1177_23743735251314620 for Understanding Patient Registries for Diabetes: A Scoping Review of Published Literature by Lana Moayad, Paige Alliston, Saira Khalid, Donna Fitzpatrick-Lewis, Hertzel C. Gerstein and Diana Sherifali in Journal of Patient Experience
