# Supplementary material for: Understanding Patient Registries for Diabetes: A Scoping Review of Published Literature
Source: J Patient Exp. 2025 Jan 21;12:23743735251314620. doi: 10.1177/23743735251314620 (PMC11748152; doi:10.1177/23743735251314620)
Supplement: sj-docx-1-jpx-10.1177_23743735251314620 - Supplemental material for Understanding Patient Registries for Diabetes: A Scoping Review of Published Literature [file sj-docx-1-jpx-10.1177_23743735251314620.docx]

Supplementary Table 1

| **Author(s), Year** | **Country of Origin** | **Healthcare Setting** | **Aim of Study** | **Aim of Registry** | **Sample Size** | **Inclusion/**  **Exclusion** | **Study Design** | **Registry Methodology** |
| --- | --- | --- | --- | --- | --- | --- | --- | --- |
| Eckert et al, 2022 | Germany | 300 diabetes centers | To evaluate changes in BP, serum lipids, physical activity, and smoking behavior in young individuals with T1D during the COVID-19 pandemic in Germany compared to previous years, based on data from a German diabetes patient registry | NR | Registry from 300 German Centers: 32,785  Total Sample in diabetes centers from Germany, Austria, Switzerland, and Luxembourg: 644,720 | Inclusion: electronic health record developed at the Institute of Epidemiology and Medical Biometry, Ulm University, Germany | Retrospective cross-sectional | Funding source of registry: German Federal Ministry for Education and Research within the German Centre for Diabetes Research (DZD, 82DZD14E03); German Robert Koch Institute (RKI); German Diabetes Association (DDG); German Diabetes Foundation (DDS, FP-0446-2022) |
| Goldfracht et al, 2011 | Israel | Primary care clinics/hospitals in Clalit | To describe the effects of a long-term intervention including 72% of Israeli diabetes patients, aimed at improving diabetes care in a primary care setting | The main infrastructure changes were allowing primary care physicians to refer patients for hemoglobin A1c (HbA1c) and low-density lipoprotein, inserting standardized follow-up forms for diabetes patients, establishing a computerized diabetes registry (first at the clinic level and later at the national level), and developing standardized reports regarding the quality of diabetes follow-up and control within the electronic medical record.  In order to evaluate changes in the follow-up and control of diabetes patients in Clalit, we compared process and outcome measures among Clalit’s members with diabetes, to diabetes patients enrolled in a different health maintenance organization (LHS). A similar registry of diabetes patients was also developed in LHS in order to facilitate reporting to the National Programme for Quality Indicators of Health in Israel. | Clalit Health Services: 3 936 800  LHS: 692 500 | Inclusion: Diabetes patients in Clalit | Retrospective cross-sectional | NR |
| Erdman et al, 2002 | USA | Urban outpatient diabetes clinic | To examine changes in lipid profiles in urban African Americans who attended a structured diabetes care program | NR | 345 | Inclusion: Included in a computerized registry; had a 1-year (52 + 10 weeks) follow-up visit; if serum total cholesterol, HDL cholesterol, LDL cholesterol, and triglyceride levels were measured at both the initial (baseline) and 1-year visits. | Retrospective cross-sectional | NR |
| Aziz, Riddell, Absetz, Brand, & Oldenburg, 2018 | Australia | Community-based (support groups) | To evaluate the implementation of a cluster randomised controlled trial of a group-based, peer support program to improve diabetes self-management and thereby, diabetes control in people with Type 2 Diabetes in Victoria, Australia | Administered by Diabetes Australia, NDSS is an initiative of the Australian Government for providing diabetes-related information and support services to people living with diabetes | 273 | NR | Cluster randomised controlled trial | NR |
| Naik et al, 2012 | USA | Primary care | To evaluate the comparative effectiveness of two diabetes group clinic interventions on glycosolated hemoglobin (HbA1c) levels in primary care | NR | 87 | NR - The registry was used as a recruitment method for the RCT | Randomized Clinical Trial | NR |
| Brazeau et al, 2022 |  | Community-based setting (17 administrative areas in Quebec) | To describe the baseline characteristics of the BETTER registry cohort and provide an overview of the baseline characteristics of the 1,430 participants 14 years of age enrolled as of February 2021 | The BETTER (BEhaviors, Therapies, TEchnologies and hypoglycemic Risk in Type 1 diabetes) registry is a type 1 diabetes population surveillance system codeveloped with patient partners to address the burden of hypoglycemia and assess the impact of new therapies and technologies. | 1430 | Inclusion: individuals must self-report a clinical diagnosis of T1D or latent-autoimmune diabetes in adults (LADA), provide a valid address in the province of Québec (Canada) and be able to read French or English | Retrospective cross-sectional | Recruitment methods: 1) be approached by a research assistant during their follow-up appointment, 2) obtain a flyer made available in a clinic waiting room, or 3) receive a letter or e-mail as an invitation to enroll in the registry. Recruitment also relies on social media: 4) by collaborating with different associations such as Diabète Québec, Diabetes Canada, the Juvenile Diabetes Research Foundation or the Diabetic Children’s Foundation to promote enrolment, or 5) directly on Facebook groups for people withT1D. Other approaches include: 6) networks of health-care professionals, 7) presentation of the project during conferences/seminars for health-care professionals to incite them to invite their patients to participate, 8) promotion during events for patients (e.g. Juvenile Diabetes Research Foundation’s Walk to Cure Diabetes), 9) the study website (https:// type1better.com/en/home), 10) Connect1d platform (connect1d.ca) and 11) patient partners advocacy within the community.  Delivery methods: Online questionnaire; Main outcomes of the registry; Clinical vs lifestyle data; Patient reported data;  Funding source of registry: The BETTER registry is funded by Strategy Patient-Oriented Research (Grant No. JT1-157204) Network and the Juvenile Diabetes Research Foundation (Grant No. 4-SRA-2018-651- Q-R Innovative Clinical Trials Multi-Year Grant). The BETTER registry is also funded by unrestricted grants from Eli Lilly & Co, Novo Nordisk and Sanof |
| Gerber, Solomon, Shaffer, Quinn, & Lipton, 2007 | USA | NR | To describe the pilot phase of an Internet program to assist these individuals who are transitioning to adult-centered medical care | NR | 19 | NR - The registry was a recruitment method for the pilot intervention study | Pilot Study of the Internet-based transition support program (Self-management Training in Youth for Lifelong Effectiveness) | NR |
| Vachon et al, 2007 | USA | Community-based (Chicago) | To describe the development and implementation of a multifaceted program in an inner-city healthcare center designed to improve access to care and empower patients to take a more active role in managing diabetes | The web-based patient registry, the BridgingCare Planner, is used to generate a list of patients with diabetes, record patient attendance to DRIVE Day, and track clinical data to support the Austin Health Center in monitoring key diabetes indicators. | 294 | NR | Retrospective cross-sectional | Delivery methods: web-based |
| Sherifali, Greb, Amirthavasar, Gerstein, & Gerstein, 2011 | Canada | Community-based | To describe and determine the impact of Diabetes Hamilton (DH), a novel, voluntary, community-based programme in Hamilton, Ontario, that aims to facilitate self-management behaviours by supplementing existing resources | DH’s goals have been to: (a) supplement the limited diabetes specialty services and tools by identifying, developing and providing community-based resources; (b) promote and facilitate diabetes self-management by a broad range of health care providers; (c) create a ‘diabetes friendly’ community; and (d) sensitise the city in general to the growing diabetes epidemic | Individuals: 3161; Family physicians: 550 | Inclusion: people with diabetes and health care providers in the Hamilton area | Retrospective cross-sectional | Recruitment methods: Personal invitations, multi-media promotion (TV), radio and newspaper, public education events;  Delivery Methods: In-person, web-based;  Main outcomes of the registry: Clinical vs lifestyle data; Patient reported data; Frequency of data collection and reporting: Baseline, annually;  Funding source of registry: Educational grants from Sanofi-Aventis, Merck Frosst and Novo Nordisk) |
