# Supplementary material for: Understanding Patient Registries for Diabetes: A Scoping Review of Published Literature
Source: J Patient Exp. 2025 Jan 21;12:23743735251314620. doi: 10.1177/23743735251314620 (PMC11748152; doi:10.1177/23743735251314620)
Supplement: sj-docx-2-jpx-10.1177_23743735251314620 - Supplemental material for Understanding Patient Registries for Diabetes: A Scoping Review of Published Literature [file sj-docx-2-jpx-10.1177_23743735251314620.docx]

**Supplemental Appendix 1: Search Strategy**

Ovid MEDLINE

1. exp diabetes mellitus/
2. diabetes.mp.
3. exp patient participation/
4. patient led.mp.
5. registries/
6. patient registry.mp.
7. 1 or 2
8. 3 or 4
9. 5 or 6
10. 7 and 8 and 9

EMBASE

1. exp diabetes mellitis/
2. diabetes.mp.
3. exp register/
4. exp patient registry/
5. registr.mp.
6. exp patient participation
7. patient led.mp.
8. 1 or 2
9. 3 or 4 or 5
10. 6 or 7
11. 8 and 9 and 10

EMCARE

1. exp diabetes mellitus/
2. diabetes.mp.
3. exp patient registry/
4. patient registry.mp.
5. registry.mp
6. 1 or 2
7. 3 or 4 or 5
8. 6 and 7

Pubmed

1. exp diabetes mellitus/
2. diabetes.mp
3. exp patient registry/
4. registr.mp
5. 1 or 2
6. 3 or 4
7. 5 and 6
